# Towards Achieving Equity and Innovation in Newborn Screening across Europe

**DOI:** 10.3390/ijns8020031

**Published:** 2022-05-06

**Authors:** Jaka Sikonja, Urh Groselj, Maurizio Scarpa, Giancarlo la Marca, David Cheillan, Stefan Kölker, Rolf H. Zetterström, Viktor Kožich, Yann Le Cam, Gulcin Gumus, Valentina Bottarelli, Mirjam van der Burg, Eugenie Dekkers, Tadej Battelino, Johan Prevot, Peter C. J. I. Schielen, James R. Bonham

**Affiliations:** 1 Department of Endocrinology, Diabetes, and Metabolic Diseases, University Children’s Hospital, University Medical Centre Ljubljana, Bohoričeva ulica 20, SI-1000 Ljubljana, Slovenia; jaka.sikonja@gmail.com (J.S.); tadej.battelino@mf.uni-lj.si (T.B.); 2 Faculty of Medicine, University of Ljubljana, Vrazov trg 2, SI-1000 Ljubljana, Slovenia; 3 Regional Coordinating Center for Rare Diseases, European Reference Network for Hereditary Metabolic Diseases (MetabERN), Udine University Hospital, Piazzale Santa Maria della Misericordia 15, 33100 Udine, Italy; maurizio.scarpa@metab.ern-net.eu; 4 Department of Experimental and Clinical Biomedical Sciences, University of Florence, 50139 Florence, Italy; giancarlo.lamarca@meyer.it; 5 Newborn Screening, Clinical Chemistry and Pharmacology Lab, Meyer Children’s University Hospital, 50139 Florence, Italy; 6 Department of Biochemistry and Molecular Biology, Groupement Hospitalier Est, Hospices Civils de Lyon, 59 Boulevard Pinel, CEDEX, 69677 Bron, France; david.cheillan@chu-lyon.fr; 7 Division of Child Neurology and Metabolic Medicine, Center for Child and Adolescent Medicine, Heidelberg University Hospital, Im Neuenheimer Feld 430, 69120 Heidelberg, Germany; stefan.koelker@med.uni-heidelberg.de; 8 Center for Inherited Metabolic Diseases, Karolinska University Hospital, SE-171 76 Stockholm, Sweden; rolf.zetterstrom@regionstockholm.se; 9 Department of Molecular Medicine and Surgery, Karolinska Institutet, SE-171 76 Stockholm, Sweden; 10 Department of Pediatrics and Inherited Metabolic Disorders, Charles University-First Faculty of Medicine, 12808 Prague, Czech Republic; viktor.kozich@vfn.cz; 11 General University Hospital in Prague, Ke Karlovu 2, 12808 Prague, Czech Republic; 12 EURORDIS-Rare Diseases Europe, 75014 Paris, France; yann.lecam@eurordis.org (Y.L.C.); gulcin.gumus@eurordis.org (G.G.); valentina.bottarelli@eurordis.org (V.B.); 13 Laboratory for Pediatric Immunology, Department of Pediatrics, Willem-Alexander Children’s Hospital, Leiden University Medical Center, Albinusdreef 2, 2333 ZA Leiden, The Netherlands; m.van_der_burg@lumc.nl; 14 Centre for Population Research, National Institute for Public Health and the Environment (RIVM), 3720 BA Bilthoven, The Netherlands; eugenie.dekkers@rivm.nl; 15 International Patient Organisation for Primary Immunodeficiencies, Downderry, Cornwall PL11 3LY, UK; johan@ipopi.org; 16 Office of the International Society for Neonatal Screening, Reigerskamp 273, 3607 HP Maarssen, The Netherlands; peter.schielen@gmail.com; 17 Sheffield Children’s NHS Foundation Trust, Western Bank, Sheffield S10 2TH, UK

**Keywords:** newborn screening, NBS, rare diseases, access inequality, Europe, Slovenia, meeting

## Abstract

Although individual rare disorders are uncommon, it is estimated that, together, 6000+ known rare diseases affect more than 30 million people in Europe, and present a substantial public health burden. Together with the psychosocial burden on affected families, rare disorders frequently, if untreated, result in a low quality of life, disability and even premature death. Newborn screening (NBS) has the potential to detect a number of rare conditions in asymptomatic children, providing the possibility of early treatment and a significantly improved long-term outcome. Despite these clear benefits, the availability and conduct of NBS programmes varies considerably across Europe and, with the increasing potential of genomic testing, it is likely that these differences may become even more pronounced. To help improve the equity of provision of NBS and ensure that all children can be offered high-quality screening regardless of race, nationality and socio-economic status, a technical meeting, endorsed by the Slovenian Presidency of the Council of the European Union, was held in October 2021. In this article, we present experiences from individual EU countries, stakeholder initiatives and the meeting’s final conclusions, which can help countries attempting to establish new NBS programmes or expand existing provision.

## 1. Introduction

From Robert Guthrie’s first description of newborn screening (NBS) for the detection of phenylketonuria in the 1960s, to the widespread use, sixty years later, of tandem mass spectrometry (MS/MS), NBS has evolved from the detection of a single disease by the use of a specific test to approaches that can simultaneously detect more than 40 conditions [1,2]. In the near future, use of genomic testing performed shortly after birth may offer the opportunity to broaden screening to several hundred conditions depending upon how this is applied. During this time, NBS has been recognised as one of the greatest advances of modern public health medicine [3], particularly in the field of rare diseases. Despite the rarity of these individual diseases, the cumulative burden of more than 6000 identified rare conditions—inherited metabolic diseases forming the largest group with about 1600 diseases (www.iembase.org, accessed on 17 February 2022)—is substantial. It has been estimated that among 750 million Europeans (at least 30 million individuals) are affected by one of over 6000 rare diseases [4,5]. Furthermore, although some disorders that are screened for (e.g., glucose-6-phosphate dehydrogenase deficiency, congenital hearing loss, congenital heart defects) are not considered rare, they may affect additional 40 million individuals [6,7,8].

For some rare disorders, the early detection offered by NBS can be life-changing and is able to prevent long-term disability or even death [9]. In the EU, it is estimated that over 4000 children each year currently benefit from screening. Expansion of NBS programmes is driven by technological advances and improved accessibility of novel diagnostic technologies (e.g., MS/MS and molecular technologies), emerging therapeutic options (e.g., gene therapy and enzyme replacement therapies) and also by improved knowledge of rare disorders [10]. However, European countries have taken significantly different attitudes to NBS implementation [11]. This can, in part, be explained by the level and type of evidence required to support screening, the need, in some countries, to demonstrate cost-effectiveness and the make-up of the national decision making bodies who determine if a new disorder is to be added to the national panel [9,12]. Direct interest or intervention at a political level can also be influential within individual member states.

To help understand and optimise the potential offered by NBS and the safeguards that must be in place to mitigate the unforeseen consequences of whole population screening programmes, the Slovenian Presidency of the Council of the European Union 2021 endorsed a technical meeting on “Achieving Equity and Innovation in Newborn Screening and in Familial Hypercholesterolemia Paediatric Screening across Europe” and listed it among its accompanying events. The technical meeting, that took place on the 11th of October 2021, brought together leaders in the field, patient representatives, members of the European Reference Networks for Rare Diseases and interested policymakers. The event was attended by more than 150 participants from more than 40 countries around the globe. As many could not attend the meeting, recordings of presentations have been made public at: https://youtube.com/playlist?list=PLGrPQqFNXCItYEmEuM7MCrbZnYGd5yCMI, (accessed on 15 March 2022).

The key objectives of the meeting were to: (i) understand and celebrate what has already been achieved within Europe in the field of NBS; (ii) understand the differences that exist in approaches to NBS and seek to improve the equity of provision between member states, taking care to respect national autonomy and subsidiarity; (iii) identify areas of good practice, helping to ensure that good practice becomes common practice; (iv) lay foundations for future development including the potential offered by genomic technologies both to detect and treat rare disorders; (v) propose the creation of a dedicated expert forum at EU level to bring together policy makers, patient group representatives and professionals able to share experience, explore the options and offer impartial advice.

In the following article, we present an executive summary of the event and its conclusions. To begin, national representatives presented examples of best practice and achievements in the field of NBS, followed by the contributions from patient advocacy organisations and pan-European initiatives. The meeting concluded with a broad discussion about the topic, leading to conclusions for further action.

## 2. An Overview of the Current State of NBS in the EU

While NBS is believed by parents to serve as reassurance that all is well, paradoxically, for a small group of parents the consequence of screening is that they are contacted unexpectedly to arrange a clinical referral following a positive NBS result. This can itself cause great shock. The way in which that clinical referral is arranged and conducted is crucially important to avoid lasting damage to the family, particularly if the result is subsequently found to be a false positive. It is clearly recognised that “all screening programmes do harm; some do good as well, and, of these some do more good than harm at reasonable cost” [13]. It is therefore important to acknowledge this at the outset so that we might act cautiously when planning new screening programmes and work tirelessly to improve the quality of those that already exist.

The International Society for Neonatal Screening (ISNS) strives to promote well-organised and carefully monitored NBS programmes that are linked to carefully planned confirmatory testing, best practice treatment and the comprehensive assessment of long-term outcome. However, in this field, significant differences can be seen between European countries. Not only do countries differ in the number of conditions screened, but also in the pre-screening information and support offered to parents, the time of sample collection, the accreditation status of the laboratories conducting screening and the governance, regulation and monitoring of the NBS pathway [11]. This variety of practice offers an opportunity to compare approaches and identify those that work well and, on that basis, help ensure that good practice becomes common practice throughout Europe. A more detailed and consistent insight into the topic can be acquired from the systematic overview of the key parameters of implemented NBS programmes and of differences between them. This was performed in 2020 for 51 European countries [11].

We are now progressing into a genetic era where genomics will help us diagnose rare disorders and also provide us with new treatment opportunities. Along with this new potential, it is important to reinforce the long-recognised importance of the Wilson and Jungner criteria [14,15,16] and in particular to ensure that: (i) we understand the natural history of the condition, (ii) the screening test is acceptable to the population, (iii) treatment is available which significantly improves the child’s long-term outcome and (iv) that the cost of case-finding (including diagnosis and treatment of patients diagnosed) should be economically balanced in relation to possible expenditure on medical care as a whole. 

While the value of the Wilson and Jungner criteria has stood the test of time, it may be that these need to be interpreted differently with the additional information made available by genomic testing [12,14,17]. Moreover, genomic testing that has the potential to significantly increase the number of conditions screened if implemented as a first-tier test will also bring many unprecedented ethical challenges. One of these is the abundance of information from genome testing and the identification of pathogenic genetic variants (and many variants of unknown significance) that do not cause an early onset disease but are rather associated with a greater risk for a certain disorder later in life.

As new technologies are emerging, including genomic testing, we will need to be cautious and ensure that the public are involved during the planning and implementation. In addition, the current COVID pandemic, which affected most of the NBS programmes in Europe at least to some degree, has clearly exposed the need for better contingency planning and sharing of best practices among countries [18].

## 3. Best Practice Models and Their Distribution

### 3.1. Italy

- Law mandate for NBS
- NBS infrastructure reorganization
- Same-day, second-tier testing results

NBS is considered an essential public health service and became mandatory by law in 1992 when only three disorders were screened for. With the introduction of MS/MS in 2016 and as new rare diseases were added, Italy began to cover approximately half of the neonatal population with expanded NBS. To improve accessibility, Italy structurally reorganized their strategy and reduced the number of screening centres from 32 to 15 to centralize diagnostic methods. Currently, the national programme includes 40 disorders, the coverage is 100% and the programme is financed by the government by Law 167/2016. To prevent parental anxiety arising from false-positive results, second-tier biochemical testing, where possible, is performed on the same day as the first screen-positive result.

### 3.2. France

- NBS mandated by law
- Clear hierarchical structure of advisory organs to the Ministry of Health

France began NBS in 1968. At present, France screens for six disorders as part of blood spot screening and clinically for hearing disability. NBS is mandatory, resulting in >99.5% coverage of newborns. The programme is performed in 17 NBS regional centres and monitored by a national coordinating centre. Two specialized commissions oversee the national organisation: an epidemiological commission which supervises the overall effectiveness of the NBS programme and a biological commission which defines the screening algorithms and proposes and monitors the biomarkers used and their cut-offs. Finally, a national steering committee in the French Ministry of Health provides the general policies for NBS. Recently, the French health technical agency recommended extending the NBS programme to include seven new inborn errors of metabolism and a new expansion is planned before the end of 2022 [19].

### 3.3. Slovenia

- Confirmatory testing with next generation sequencing
- Nationwide IT system for NBS

The first major expansion of NBS in Slovenia was implemented in 2018 with the introduction of MS/MS, when 17 inborn errors of metabolism were added to phenylketonuria and congenital hypothyroidism. Next generation sequencing was introduced for confirmatory testing of positive first-tier results and proved to be feasible providing a reliable result. In addition, every Slovenian newborn is screened for hearing impairment by transient evoked otoacoustic emissions, optic media translucency and congenital hip dysplasia by an ultrasound. Law mandates NBS, and over 99% of newborns have their blood samples analysed in a single central laboratory. Following scientific advances, a second expansion of NBS is planned for 2022 and this will be able to detect more than 40 congenital diseases. To minimize the administrative burden and to achieve good traceability and rapid reporting of results, a national IT network was established for NBS together with a barcode system [20,21]. Slovenia is also spearheading an initiative in the field of screening for familial hypercholesterolemia, one of the most common inherited disorders, which is currently not performed in the context of NBS. Nationwide cholesterol measurements are performed at the age of 5 years, followed by a next generation sequencing of hypercholesterolemia-causing genes. Screening during childhood detects children before the manifestation of cardiovascular disease and provides a basis for cascade screening of siblings and parents [22].

### 3.4. Germany

- Evaluation of feasibility of NBS extensions with pilot studies
- Long-term observational studies of children with inherited metabolic diseases
- National cohorts for individual metabolic diseases

Germany began an NBS programme in 1969 and since then has successively extended the screening panel. Since 2005, the NBS programme has been coordinated through a national directive. At present, the disease panel includes 19 diseases. In addition, regional pilot studies on additional diseases are conducted to evaluate feasibility, diagnostic process quality, and potential health benefits [23,24]. In southwest Germany, long-term observational studies of children with inherited metabolic diseases started with the birth cohort of 1999 and recently established national cohorts for individual metabolic diseases [25,26,27]. The general overview of the study findings is that NBS is coupled to appropriate treatment results in the optimum neurocognitive development. However, this does not apply to every disorder in the same way, highlighting the need to assess the clinical outcomes for each individual disorder separately.

### 3.5. Sweden

- National biobank for dried blood spots storage
- National registry for inborn errors of metabolism for prospective management of patients

Sweden began newborn blood spot screening in 1965 and has, since 1975, stored all dried blood spots in a biobank which helps support quality control of the screening programme and provides a basis for research. At present, 25 disorders are included in the screening programme, for some a second-tier test is performed to improve the positive predictive value [28]. At present four paediatric metabolic centres and three paediatric centres for primary immunodeficiences receive screen-positive referrals. For the endocrine disorders congenital hypothyroidism and congenital adrenal hyperplasia, children are referred to paediatric centres in Sweden guided by a specialist also working in the NBS laboratory. In 2013 a national registry for inborn errors of metabolism was implemented. This registry is used to assess outcomes at the population level while also acting as an electronic patient medical record helping clinicians to monitor treatment and record progress [29].

### 3.6. Czech Republic

- NBS extension preceded by a pilot programme
- Determination of population-specific cut-offs
- Cost-effectiveness analyses prior to implementation

Screening for phenylketonuria began in the former Czechoslovakia in Czechia in 1975 and in Slovakia in 1980. The introduction of congenital hypothyroidism began in 1985. Subsequent expansions of the laboratory NBS programme took place in the independent Czech Republic in 2006, 2009 and 2016. These were each preceded by pilot programmes funded from competitive grant calls. The investigator-initiated pilots aimed at obtaining population distribution of the biomarkers, defining cut-off values and establishing patient care pathways and algorithms for patient care. The recommendations of these pilot projects together with estimation of costs of screening and therapy were mandated by the Ministry of Health and insurance companies prior to approving nationwide expansions. Currently, 15 inherited metabolic disorders, two endocrinopathies and cystic fibrosis are the primary target conditions; LC-MS/MS and molecular genetic techniques are used as second-tier tests for selected inborn errors of metabolism and cystic fibrosis, respectively. A new pilot programme for spinal muscular atrophy and severe combined immunodeficiency, initiated by policy makers, a patient organisation and healthcare professionals, began in January 2022.

### 3.7. Southeastern Europe—An Underdeveloped Region

- Generally underdeveloped NBS programmes compared to Western countries
- No NBS programmes in two countries in 2020

Southeastern Europe comprises 14 diverse countries, 7 of them are EU member states. NBS programmes in the region were generally underdeveloped when compared with western European countries in 2013. At the time, none of the countries had incorporated MS/MS in the programme, with the exception of Hungary in 2007. This was later followed by Croatia in 2017, and Slovenia in 2018. Nevertheless, it is a serious concern that two countries in the region currently do not have an NBS programme available for their population and that children in these countries are deprived of this life-changing intervention. Encouragingly, in the countries with existing NBS, the number of diseases screened and the proportion of newborns included has risen steadily. The main obstacles for programme expansion are the lack of financial resources, lack of organisation and staff [30,31]. The complex and diverse situation in this region illustrates the need for a greater collaboration within the NBS community to help improve the situation in some of the less-developed countries of Europe; this might include schemes for exchange of experiences, best-practices, and professionals (e.g., training opportunities).

## 4. Roles of Key Stakeholders

### 4.1. Screen4Rare

Screen4rare is a multi-stakeholder initiative launched by the International Patient Organisation for Primary Immunodeficiencies (IPOPI), the ISNS and the European Society for Immunodeficiencies. Screen4Rare recognises the need for autonomous national policy in member states and seeks to support those with that responsibility by the provision of unbiased scientific information, evidence, and comparative data to help ensure that the best decisions can be made on behalf of the populations served. 

The primary goal of Screen4Rare is to help promote ”The development of appropriate, well-organised and equitable NBS offered on a voluntary and informed basis to families, to help identify well-defined and treatable conditions where it is clear that their early asymptomatic detection and treatment during childhood results in significantly improved outcome.” To achieve this goal, Screen4Rare is striving to stimulate debate and support member states as they seek to improve existing screening programmes and, where appropriate, to introduce new forms of screening. Screen4Rare seeks to engage with physicians and scientists working in the field together with interested politicians, health policy makers and patient/parent groups with an interest. 

The EU Screen4rare initiative has achieved many important milestones including the support of 30 Members of the European Parliament and several organisations to a Call to Action on NBS for rare diseases [32]. Screen4Rare also supported the launch of a European Reference Network Expert Platform on NBS in collaboration with the European Commission (EC), the European Reference Network for Rare Hereditary Metabolic Disorders (MetabERN) and the European Reference Network on Rare Primary Immunodeficiency, Autoinflammatory and Autoimmune Diseases (ERN-RITA) [33]. In 2021, to help raise awareness, Screen4rare launched the first International Neonatal Screening Day which will be, in the future, held every year on June 28th. To ensure that we prepare for the future and a fast-evolving NBS landscape, Screen4Rare is committed to continue supporting EU NBS actions.

### 4.2. The Views of People Living with Rare Diseases and Their Families: 11 Key Principles

EURORDIS is a non-governmental, patient-driven alliance of patient organisations representing 988 rare disease patient organisations in 74 countries. In 2019, EURORDIS set up the Newborn Screening Working Group to review current policy and practice in the field of NBS and to develop principles for harmonious uptake/adoption of the NBS programmes across the Member States with a view to delivering maximum benefit and improving outcomes for babies born with rare diseases.

After a consultation process involving experts, stakeholders as well as national alliances in the countries across Europe, European and international federations of rare diseases and members of EURORDIS, a position paper was published in January 2021 [34]. The position paper includes 11 key principles on NBS and a call to action for the harmonisation of these principles to reduce the vast disparities between countries and diseases that are included in the NBS national programmes. It calls for collaboration across the EU Member States—and potentially beyond—in areas where it could bring added value to national action and for the European Institutions to promote the sharing of best practices across countries.

The principles make it clear that NBS should be organised as a system with clearly defined roles and responsibilities, as well as governance and accountability structures that are transparent and robust. The principles advocated by EURORDIS include, but are not limited to, treatable diseases, as this may improve the overall quality of life for newborns and their families. Crucially, it avoids the long journey to diagnosis that is so often faced, allows parents to also make decisions about treatments that can prevent or slow the onset of serious symptoms and gives them the option of making informed decisions about future pregnancies. Moreover, the principles promote appropriate psychological, social and economic support standards to be in place for families whose newborn is screened. In the position paper, it is also mentioned that healthcare professionals should receive thorough training, and the need for broader public awareness. Finally, the principles propose that Europe-wide standards are established to ensure that every parent in Europe can expect the same timing, collection methods, follow-up and information.

### 4.3. European Reference Networks

In 2002, patients suffering from rare diseases reached out to the EC and raised awareness of rare diseases. This helped develop the concept of the European Reference Networks (ERNs) supported by Art. 12 in the Cross Border Directive 2011/24/EU. In 2017, twenty-four ERNs were established, involving about 300 health care providers of excellence and about 1000 specialized units, to which about 620 new units were added in November 2021. ERNs are present in all 27 EU Member States. “Share, Care, Cure” is the ERN’s motto, to promote equity of access to diagnosis, management and treatment for rare diseases in the EU. It is recognised that NBS has a key role in helping to achieve these goals and in turn, that the ERNs may have valuable expertise to inform how screening can be organized to ensure the best possible outcome for children and their families. Moreover, the ERNs seek to facilitate research and implementation of innovative therapies for rare diseases in EU. The ERNs are committed to promoting the concept that NBS should be considered as a system, not just a test, for this reason, this begins with information to couples during pregnancy and full counselling and care at the point of diagnosis [9]. 

## 5. Policy-Oriented Multi-Stakeholder Expert Advisory Committee

Equitable access to NBS—a life-changing and sometimes a lifesaving intervention—can be achieved by working together and the careful consideration of existing models of service delivery. We believe that it may be helpful to establish a time-limited NBS expert advisory committee (NBS-EAC), to help inform the EC and to act as a source for unbiased, evidence-based advice to support decision making at a national level. It is important that the group should include the voices of patient support organisations and be equipped to address the key issues of value to health-policy makers. The ERNs already have an established network of experts from different fields, connected to NBS, and as such provide an ideal platform for the formation of an NBS-EAC. Screen4Rare can also assist as a trusted repository for information about the NBS programmes across member states. The newly formed NBS-EAC would aim to develop unbiased information to support existing screening programmes and share information about new developments in all European countries.

## 6. Key Points and Future Challenges

A key role for the NBS-EAC will also be to share experience from pilot schemes taking place in individual member states. It could play a valuable role in helping to co-ordinate work streams seeking to establish consistent case definitions for conditions included within NBS programmes and help ensure the interoperability of long-term outcome studies by supporting the ongoing development of existing disease registries in this area. It would also provide a means to share experience from around Europe of the governance arrangements, including a dictionary of key performance indicators, which can be employed to assess and monitor the quality of NBS programmes along with the standards which have been set by various member states to help monitor these. 

The key points that were identified in the stakeholders’ discussions are presented in Table 1.

## 7. Urgent Initiatives

As a product of this ongoing collaboration, a recent paper describes how NBS as a fully integrated system can enhance equity in NBS in Europe. This may need ten elements for the effective operation of NBS programmes, in part overlapping with initiatives mentioned below [9].

The most urgent initiatives, needed to stabilise the existing position and plan for a future which may see rapid expansion reflecting technological change, include the following:

(i) The formation of an NBS-EAC free from bias or national interests to provide trusted, high-quality information to support decision making at a national level;
(ii) The need to progress specific work streams related to the documentation, identification and promotion of good practice in existing national NBS programmes so that these lessons may be shared more widely including through the Non-Communicable Diseases Group (SGPP) advising the EC;
(iii) The need to collect, collate and develop key performance indicators that might help maintain and improve the quality of NBS programmes;
(iv) The establishment of accepted ‘Case Definitions’ for the disorders currently screened and those under consideration;
(v) The promotion of interoperable disease registries as a means to gather and understand outcomes to guide NBS strategy;
(vi) The need for outputs from national pilot programmes in NBS to be shared more effectively to shorten the time needed to introduce new screening programmes or cease their development where these may be shown to be inappropriate;
(vii) The consolidation of a NBS group within the existing ERNs, both those with a current involvement in NBS such as MetabERN and ERN-RITA (the immune deficiency ERN), and in those who have responsibility for conditions where patients may benefit from the early asymptomatic detection offered by NBS in the near future;
(viii) The need for special consideration to be given to the rapid development of genomics to greatly alter the potential for diagnosis at birth and the ethical challenges and clinical opportunities that this brings.

## 8. Conclusions

It was recognized at the meeting that NBS enjoys widespread acceptance among the public, professionals and policy makers and great care must be taken to preserve this public confidence in a society which no longer accepts medical practice without question. We need to be able to respond to those requesting improved access to screening while acting proportionately and ethically to protect the confidence which the public and parents with young children have placed in our existing programmes. To achieve this equitably on behalf of the community that we serve in the EU, we need to work together collaboratively across member states with the backing of the EC and the support of politicians, parents, professionals and the public. Additional initiatives are needed to promote good practice, address inequity and to ensure that the EU can maximise the scientific, clinical and economic benefits arising from NBS in the coming years.

## Figures and Tables

**Table 1:** The key points identified in stakeholders’ discussion. Abbreviations: NBS—Newborn screening; ERN—European Reference Networks.

The Key Points Identified in Stakeholders’ Discussion.
(1) To celebrate and promote newborn screening (NBS) as a life-changing intervention for children with rare disease, helping to ensure a good clinical outcome in serious or life-threatening conditions.
(2) The considerable unwarranted variation in practice, both in the number of conditions screened at birth, ranging from 2 to 35 per country, and in the way in which NBS is planned and delivered.
(3) The use of second-tier testing t avoid false-positive results that may unnecessarily alarm families.
(4) The need for clear case definitions for disorders allowing comparison and improvement of the effectiveness of NBS programmes in order to optimize treatment strategies.
(5) The importance of ensuring that the outcome of NBS programmes is assessed and used to guide current and future practice: a core role for the European Reference Networks (ERNs).
(6) The vital importance of managing national NBS as a programme spanning community, laboratory and clinical activity to ensure that outcomes are improved and good clinical practice achieved. (7) The desire to identify and monitor key performance indicators to help assess the operational effectiveness and quality
(8) The need to share information between member states as NBS programmes grow and develop.
(9) The opportunities and ethical challenges for member states posed by the increased availability of genomic testing and treatment when considered in the context of NBS for Rare Diseases including its potential to significantly increase the range and scope of conditions identified.

## Data Availability

Not applicable.

## Abbreviations

EC	European Commission
ERN	European Reference Network
ERN-RITA	European Reference Network on Rare Primary Immunodeficiency, Autoinflammatory and Autoimmune Diseases
IPOPI	International Patient Organization for Primary Immunodeficiencies
ISNS	International Society of Neonatal Screening
MetabERN	European Reference Network for Hereditary Metabolic Disorders
MS/MS	tandem mass spectrometry
NBS	newborn/neonatal screening
NBS-EAC	NBS expert advisory committee
SGPP	Steering Group on Health Promotion, Disease Prevention and Management of Non-Communicable Diseases
